# Novel Polysaccharide Hydrogels Enriched with Humic Acid for Sustainable Agricultural Applications

**DOI:** 10.1021/acsomega.5c09496

**Published:** 2025-12-16

**Authors:** Ana V. Torres-Figueroa, Sergio de los Santos-Villalobos, Dora E. Rodríguez-Félix, Gerardo Valenzuela-Hernandez, Sergio F. Moreno-Salazar, Cinthia J. Pérez-Martínez, Andrés Ochoa-Meza, Teresa del Castillo-Castro

**Affiliations:** † Departamento de Investigación en Polímeros y Materiales, 27813 Universidad de Sonora, Hermosillo 83000, Mexico; ‡ Laboratorio de Biotecnología Del Recurso Microbiano, Departamento de Ciencias Agronómicas y Veterinarias, 27745 Instituto Tecnológico de Sonora, 5 de Febrero 818 Sur, Colonia Centro, Ciudad Obregón 85000, Mexico; § Departamento de Agricultura y Ganadería, Universidad de Sonora, Carr. Bahía de Kino, Km. 21. Apartado Postal 305, Hermosillo 83323, Sonora, Mexico; ∥ Departamento de Ciencias Químico Biológicas, Universidad de Sonora, Hermosillo 83000, Mexico; ⊥ Departamento de Investigación en Física, Universidad de Sonora, Hermosillo 83000, Mexico

## Abstract

This study deals with the development of humic acid (HA)-enriched hydrogels aimed at enhancing water retention and promoting plant growth for agricultural applications. A set of hydrogels based on spermidine (SPD)-ionotropically cross-linked gellan gum (GG) were formulated with and without the addition of karaya gum (KG). The hydrogels were structurally characterized by Fourier transform infrared spectroscopy, thermogravimetric analysis, mechanical assays, scanning electron microscopy, and swelling kinetic measurements. Water retention tests indicated that both GG/HA and GG/KG/HA hydrogels preserve higher soil moisture levels compared to commercial polyacrylate hydrogels. Biodegradation studies showed that the biopolymer hydrogels lost more than one-third of their weight after 30 days of immersion in a soil aqueous extract. Both GG/HA and GG/KG/HA hydrogels exhibited no phytotoxicity on cultivation trials of *Triticum aestivum* L. var. Borlaug 100, but the plant growth was promoted with the GG/KG/HA network compared to GG/HA sample. The overall results support the potential use of GG/KG-based hydrogels enriched with HA as sustainable materials for improving soil quality and crop productivity in agricultural systems.

## Graphical abstract

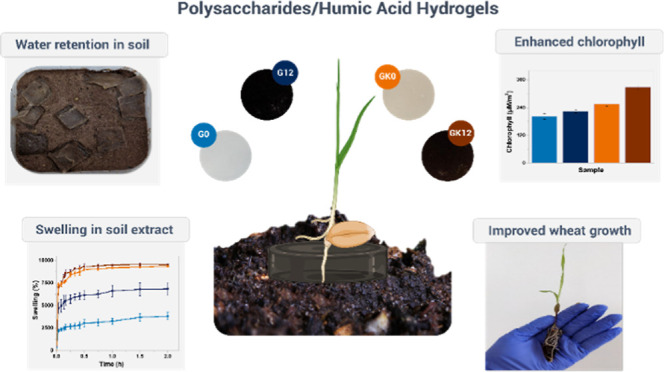

## Introduction

Global food production faces unprecedented challenges due to climate change, land degradation, and a rapidly growing population. By 2050, food production must rise significantly to meet global demand while preserving soil health and ecosystem stability.^1,2^ One of the most pressing limitations is water scarcity, particularly in arid and semiarid regions, where irregular precipitation and high evapotranspiration reduce water availability for crops.^3^ In these settings, improving water use efficiency is essential to support sustainable and productive farming.^4^

Among emerging solutions, hydrogels, that are three-dimensional hydrophilic polymer networks capable of absorbing and retaining large volumes of water, have shown promise as soil conditioners.^5,6^ These materials can reduce irrigation frequency, buffer crops against drought, and improve soil texture and aeration.^4^ However, the most widely used hydrogels in agriculture are based on synthetic polymers such as poly­(acrylic acid) or poly­(acrylamide), which raise environmental concerns due to their limited biodegradability and potential toxicity of their degradation products.^2,7,8^ As a consequence, increasing interest has been directed toward biodegradable and biocompatible alternatives derived from natural polysaccharides, which are abundant, renewable, and economically sustainable sources for hydrogel fabrication.^9,10^

Gellan gum (GG) is an FDA-approved food additive due to its biocompatibility and biodegradability, characteristics that also make it a promising material for agricultural applications.^8,11–13^ This anionic extracellular microbial polysaccharide contains tetrasaccharide repeating units consisting of d-glucuronic acid, l-rhamnose, and two d-glucose units. Also commercially known as phytagel, GG is commonly utilized as a substitute for agar in agricultural media for growing plant tissues.^14^ In addition, bacteria encapsulated in chitosan and GG microcapsules have been used for controlling take-all disease of wheat.^15^ Moreover, poly­(acrylic acid) hydrogels copolymerized with GG have been proposed for agricultural applications because of their water retention capacity.^2^ Furthermore, the incorporation of konjac-glucomannan in GG hydrogels has improved the germination and chlorophyll content of fenugreek microgreens under semiarid conditions.^16^

The functionality of polysaccharide-based hydrogels depends greatly on the nature of their cross-linking.^12,17^ Since GG (p*K*_a_ ≈ 3.5) is anionic in neutral water, gelation is typically achieved by adding suitable cationic species to form stable hydrogel networks. Spermidine (SPD), a naturally occurring cationic polyamine containing three protonatable amine groups (p*K*_a_ ≈ 8.5, 9.8, 10.8), has been successfully used for cross-linking GG.^12^ Due to the phytohormonal regulatory effects of polyamines, SPD has been employed as a stress protector in plants. For instance, in wheat, SPD increased drought tolerance during the germination stage due to the specific role of polyamine metabolism in developing effective responses under drought stress.^18,19^ SPD-cross-linked hydrogels thus provide a bioactive network architecture that may enhance both structural performance and plant response.

Another polysaccharide with unique features is karaya gum (KG), a partially acetylated acidic exudate primarily composed of galacturonic acid, β-d-galactose, glucuronic acid, l-rhamnose, and other residues.^20^ KG is biodegradable, nontoxic, and capable of forming viscous gels with high swelling capacity. Recently, it has also been used as a gelling agent in place of agar for the micropropagation of rough lemon (*Citrus jambhiri* Lush.).^21^

On the other hand, the incorporation of soil conditioners or plant biostimulants into hydrogels intended for agricultural applications has gained attention.^22^ Humic acids (HA) derived from the decomposition of organic matter, are widely recognized for improving soil fertility, enhancing nutrient uptake, and stimulating plant metabolism.^23,24^ The combination of soil and foliar application of HA in Mexican lime trees, *Citrus aurantifolia* (Christm.) has shown great potential for alleviating the effects of salinity stress on growth, productivity, and fruit quality.^24^ Additionally, the application of HA in wheat crops has increased grain size and weight in greenhouse experiments.^25,26^

Despite these advances, no previous studies have reported a hydrogel system that simultaneously incorporates GG, KG, and HA cross-linked with SPD for agricultural use. The integration of these biopolymers into a single hydrogel platform may result in a multifunctional material with enhanced swelling behavior, biodegradability, and plant growth-promoting effects. Such properties are particularly important for sustainable agricultural soils and provide a convenient alternative to synthetic hydrogels.

In this regard, this study aims to develop a novel multifunctional material for agricultural purposes, particularly to support wheat growth. Wheat (*T. aestivum* L) is one of the most important cereals cultivated globally for food and nutritional security.^27^ However, wheat yield faces numerous challenges, including water deficit, which poses a serious threat to production in arid and semiarid regions of the world.^18,28^ To the best of our knowledge, this is the first study to explore this approach, yielding promising results.

Hydrogel formulations of GG/HA and GG/KG/HA with varying HA content were prepared and characterized using Fourier transform infrared spectroscopy (FTIR), thermogravimetric analysis (TGA), compression testing, scanning electron microscopy (SEM), swelling kinetic measurements, water retention evaluation in soil, and biodegradation studies in soil aqueous extract. The effects of these hydrogels on the growth and physiological traits of wheat plants were also evaluated. The capacity of GG/HA and GG/KG/HA hydrogels to retain the soil moisture, combined with the absence of phytotoxicity, evidenced their potential for enhancing plant growth and serving as water reservoirs, particularly in arid and semiarid regions.

## Materials and Methods

### Materials

GG (Gelzan CM); KG from *Sterculia* tree; HA sodium salt, technical grade; SPD trihydrochloride, 98%, were purchased from Sigma-Aldrich. All reagents were of analytical grade and used as received without further purification. The aqueous solutions were prepared with deionized water, purified by a Milli-Q Organex system (Millipore, Molsheim, France).

### Hydrogel Preparation

GG/KG/HA hydrogels were obtained through ionotropic cross-linking of GG chains with SPD in the presence of HA and KG. A GG solution (0.5 wt %) was prepared by dissolving GG in deionized water at 55 °C for 3 h. An amount of HA powder (0 or 12 wt %) was dispersed in the GG solution and maintained at 55 °C. Then, a certain amount of a KG solution (1 wt %) was added to the GG/HA mixture and stirred for 15 min to ensure homogenization. Afterward, the SPD solution (0.05 wt %), preheated at 37 °C, was added to the polymer suspension. The mixture was stirred in a cylindrical mold to properly mix all the components, followed by cooling to room temperature (25 °C). As the temperature decreased, gelation occurred.^29^ Finally, the hydrogels were removed from the mold and dried by lyophilization in a freeze–dryer Labconco FreeZone 4.5 L. GG/HA hydrogels were also prepared using the same procedure, but without adding the KG solution.

The concentrations of GG and SPD were selected based on previously reported conditions that ensured effective cross-linking and reproducibility.^29^ The HA content (12 wt %) was chosen according to swelling and stability trends observed in HA-loaded hydrogel systems,^30^ which, in our formulation, preserved gel integrity. KG was tested at various concentrations, with 1 wt % identified as optimal, since lower values failed to form stable networks and higher fractions disrupted network formation.

Table 1 summarizes the component ratios used for each hydrogel type. The hydrogel code numeral indicates the HA wt % in dried samples. Figure 1 illustrates the preparation method of GG/KG/HA hydrogels.

**Table 1:** Feed Compositions in the Preparation of Single Hydrogels^a^

sample	GG 0.5 wt % (mL)	HA (mg)	KG 1 wt % (mL)	SPD 0.05 wt % (mL)
G0	0.71	---	---	0.43
G12	0.71	0.483	---	0.43
GK0	0.35	---	0.35	0.43
GK12	0.35	0.483	0.35	0.43

a G0 and G12 correspond to GG/HA hydrogels; GK0 and GK12 correspond to GG/KG/HA hydrogels.

**Figure 1:**
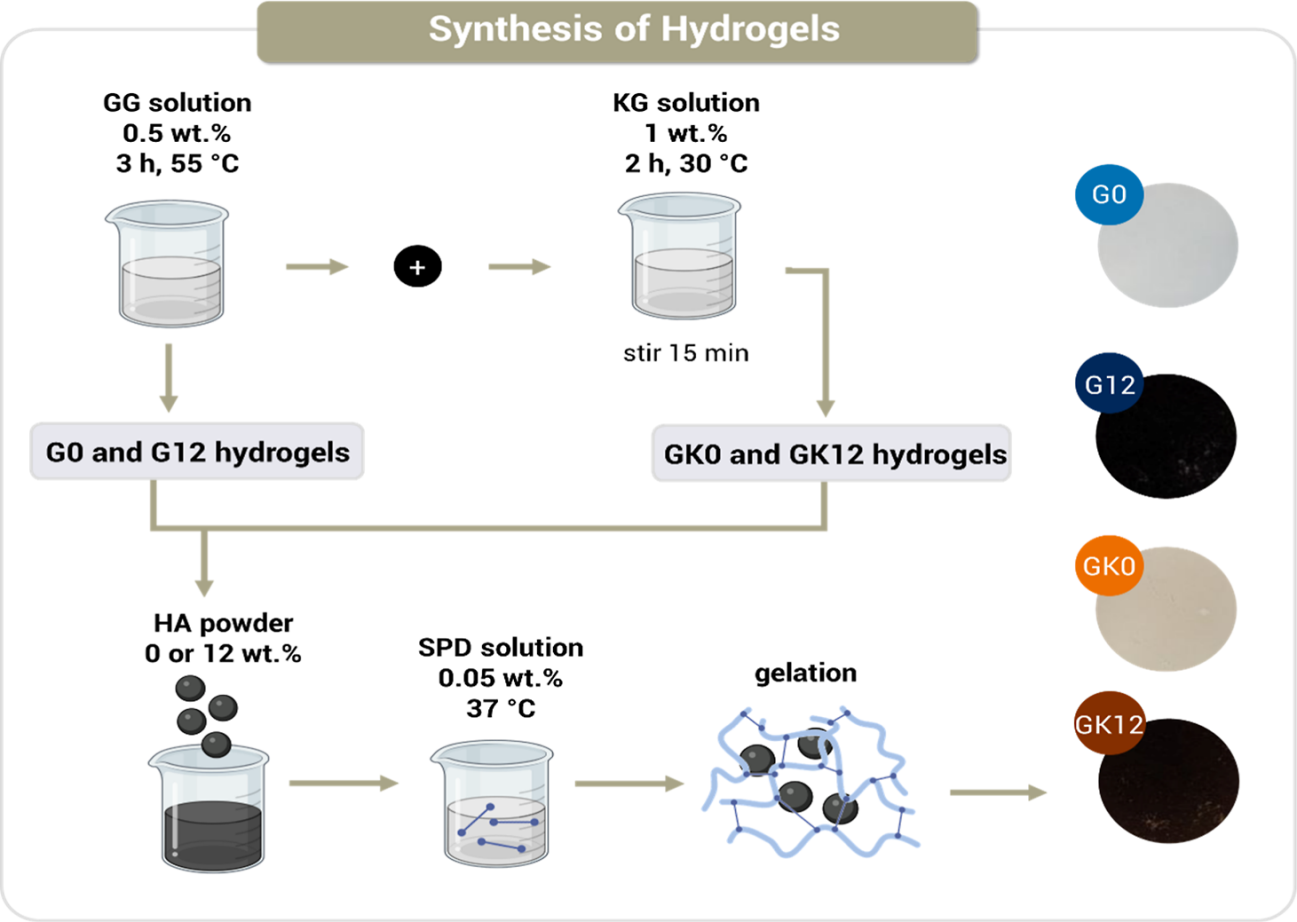
Preparation of GG/KG/HA hydrogels.

### Hydrogel Characterization

ATR-FTIR spectra were recorded in a Frontier spectrometer (PerkinElmer, Beaconsfield, UK) equipped with a single reflection diamond accessory, in a range of 4000–500 cm^–1^. TGA experiments were carried out under nitrogen flow until 600 °C and a heating rate of 10 °C min^–1^, using a Pyris 1 apparatus (PerkinElmer, Llantrisant, UK). Mechanical properties of hydrated hydrogels were evaluated in compression tests using a TA ElectroForce 5500 BioDynamic equipment with a 200 N load cell. Cylindrical-shaped hydrogels of 10 mm diameter *x* 7 mm height were compressed until deformation (rupture) at a constant strain rate of 3.5 mm s^–1^. SEM was used to study the internal morphology of hydrogel samples. The analyses were performed using a scanning electron microscope model JEOL JSM-5410LV (JEOL-LTD, Tokyo, Japan) operated with an acceleration voltage of 15 kV. Cross-sectional slices of hydrogels were frozen and lyophilized. Then, the dried samples were fixed on carbon ribbon and gold sputtered prior to SEM examination.

### Swelling Measurements

The swelling capacity of hydrogels was evaluated in deionized water and in a soil aqueous extract (pH 7.71, EC 132.9 μS cm^–1^) using the gravimetric method at 25 ± 1 °C. The soil extract was prepared according to Durpekova et al.^31^ Sterile soil (autoclaved at 120 °C and 0.12 MPa for 40 min) previously collected from the Yaqui Valley in Mexico (clay loam texture)^32^ was used for these experiments. A total of 20 g of soil was added to 1 L of deionized water, and the resultant suspension was stirred for 24 h. Afterward, the suspension was centrifuged at 3540 rpm for 10 min and the supernatant was collected for the swelling experiments.

Freeze–dried samples of known weight (*W*_0_) were immersed in the aqueous media. At specific times (*t*), the samples were removed from the swelling medium, blotted, weighed (*W*_*t*_), and placed in the same bath until constant weight was reached. Swelling measurements were performed in triplicate using dry hydrogel samples (*W*_0_ ≈ 3.2–4.8 mg for GG/HA and 6.2–7.5 mg for GG/KG/HA). Sample weights were recorded at defined intervals (0–120 min) until no further change was observed (|*W*(*t*) – *W*(*t* – Δ*t*)|/*W*(*t*) < 1%), which was taken as equilibrium. The corresponding time points are shown in the swelling curves. The swelling percentage at time *t* was calculated from the following relation (eq 1)­

1 $$swelling\left(\%\right)=\frac{W_{t}-W_{0}}{W_{0}}\times 100$$

### Water Retention Capacity

The ability of hydrogels to retain water in soil was assessed by measuring the water evaporation ratio (WER) at 25 ± 1 °C. Sterile soil collected from the Yaqui Valley was dried at 60 °C for 48 h in a mechanical convection laboratory incubator (Thermo Scientific Precision 3511, USA). Freeze–dried polysaccharide hydrogels (0.5 wt %), along with a commercial hydrogel sample (Wet Smart, potassium polyacrylate), were separately buried in 60 g (*W*_0_) of the dried soil into plastic pots. Pure soil was used as the control. Next, 60 mL of deionized water was added to each pot, and their weights registered (*W*_1_). The samples were stored at room temperature, and their weights were monitored at different times (*W*_*t*_) until no further detectable weight loss was observed.^31,33^ The WER percentage at time *t* was calculated from the following relation (eq 2)­

2 $$WER\left(\%\right)=\frac{W_{1}-W_{t}}{W_{0}}\times 100$$

### Biodegradation Test

Freeze-dried hydrogels were placed into a plastic pot containing the soil aqueous extract (pH 7.71, EC 132.9 μS cm^–1^). The samples were kept at room temperature for 30 days, and their weights were measured at 5 day intervals. The degree of degradation was determined by calculating the weight loss using the following relation (eq 3)­

3 $$weight\;loss\left(\%\right)=\frac{W_{i}-W_{f}}{W_{i}}\times 100$$

where, *W*_i_ is the initial weight of the sample prior to degradation and *W*_f_ refers to its weight after specific time intervals of biodegradation.^31^

### Effect of Hydrogel on Plant Growth

A phytotoxicity test was carried out to evaluate the effect of hydrogel on growth and physiological traits of wheat plants. The test was performed according to the methodology reported by Montesano et al.^6^ Seeds of wheat (*T. aestivum* L. var. Borlaug 100) were placed on Petri dishes containing PD agar (control) or the hydrogel samples. The dishes were incubated in a growth chamber (BJPX-A450, BIOBASE) to simulate environmental conditions typical of the Yaqui valley. Plant growth was observed after 3, 5, and 7 days. Each treatment included three Petri dishes with five seeds per dish (15 seeds in total).

After the observation period, the biometric parameters of wheat were measured. The seedlings were cut crosswise, separating the aerial part from the roots. The fresh weight of both parts was recorded, and then, the samples were dried in a mechanical convection laboratory incubator (Thermo Scientific Precision 3511, USA) for 48 h at 60 °C. Once dry, the samples were weighed, and the average dry weight of the aerial parts and roots is herein reported. Chlorophyll concentration in leaves was measured using an MC-100 chlorophyll concentration meter (Apogee Instruments, Inc., Logan, Utah, USA). For comparative purposes, analysis of variance (ANOVA) was carried out with an acceptable level of significance of *P* < 0.05, using the statistical package IBM SPSS Statistics 21.

## Results and Discussion

### FTIR Spectroscopy

Figure 2 shows the FTIR spectra of the GG/HA and GG/KG/HA hydrogels and those of their neat GG, KG, and HA components. Briefly, GG spectrum shows a broadband corresponding to the O–H vibrational stretching at 3506–3117 cm^–1^. A characteristic peak at 1015 cm^–1^ is assigned to the C–O–C stretching vibration of the sugar units.^29^ The KG spectrum also features a broadband related to the O–H vibrational stretching at 3567–3110 cm^–1^, while a distinct absorption at 1069 cm^–1^ is attributed to the C–O–C stretching vibrations of monosaccharide ring structures.^34^

**Figure 2:**
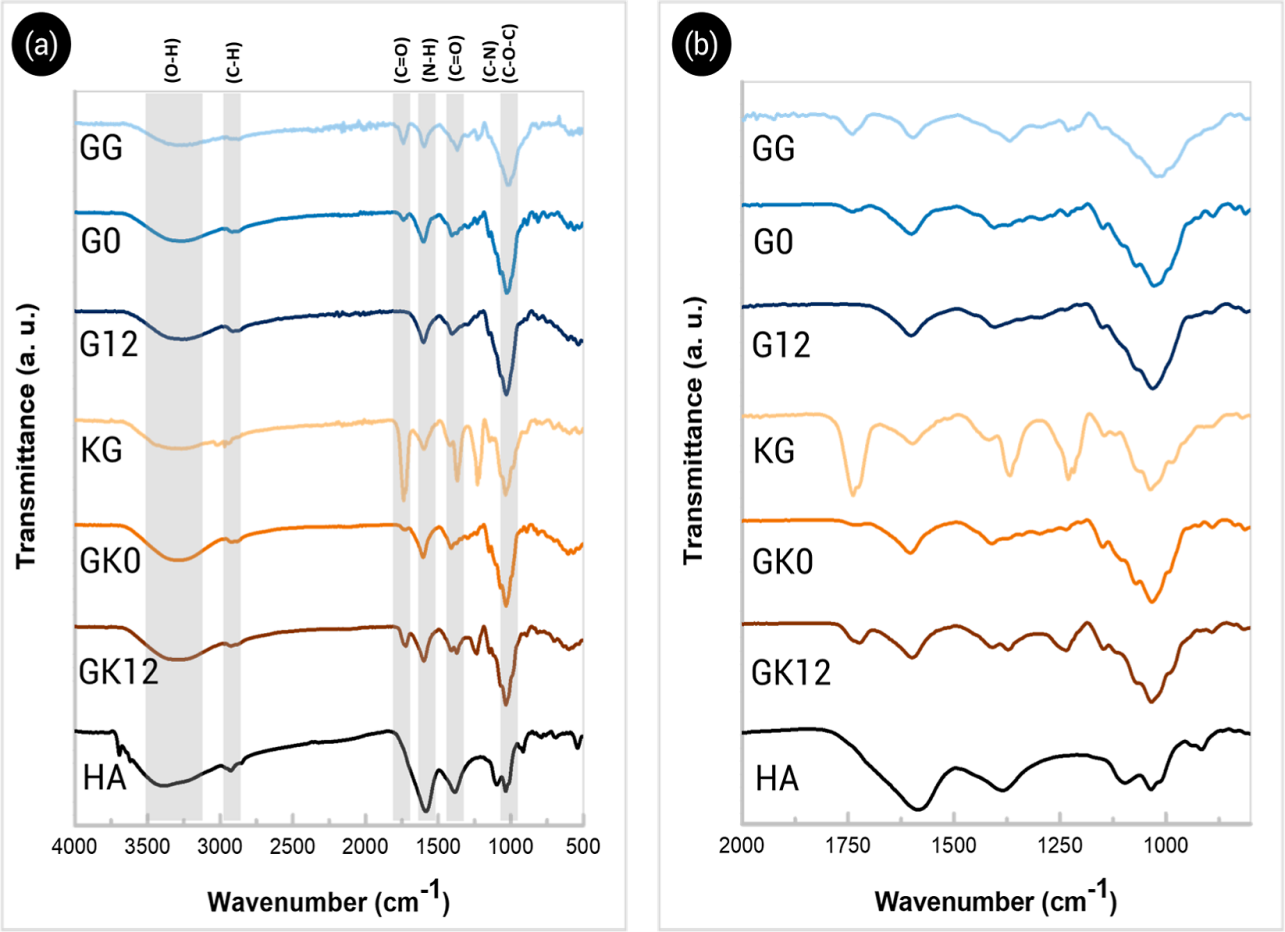
ATR-FTIR spectra of GG, KG, HA, and composite hydrogels; full spectra from 4000 to 500 cm^–1^ (a) and spectral details from 2000 to 800 cm^–1^ (b).

The HA spectrum shows a broadband at 3385 cm^–1^ attributed to the O–H vibrational stretching of the hydrogen-bonded carboxyl, alcohol, and phenol groups. The band at 2927 cm^–1^ is associated with the asymmetric C–H stretching vibration of methyl and/or methylene groups. A small shoulder at 1690 cm^–1^ is related to the COO^–^ stretching vibration, and a band at 1585 cm^–1^ is due to the C=C vibrational stretching mode. The signals at 1394 cm^–1^, 1106 cm^–1^, and 1035 cm^–1^ are attributed to COO^–^ moieties, C–O stretching vibration of phenolic groups, and C–N stretching vibrations, respectively.^23,30^

Most of the inherent absorptions of GG, KG, and HA overlap in the spectra of composite hydrogels. Overall, the bands of the hydrogel spectra are attributed to the characteristic absorption peaks of their constituents. Well-defined peaks attributed to the KG-ester bond vibrations are slightly shifted in the spectra of both GK0 (1741 and 1234 cm^–1^) and GK12 (1735 and 1235 cm^–1^) hydrogels compared to the spectrum of single KG. The bands corresponding to the SPD cross-linker are also observed; particularly, the N–H bending band close to 1615 cm^–1^ and the C–N stretching band close to 1094 cm^–1^. Although the absorption regions of GG, KG, and HA largely overlap, preventing precise resolution of individual peak shifts in the composite hydrogels, the spectra exhibit consistent trends in two key regions. The O–H stretching band (3600–3000 cm^–1^) becomes broader and slightly shifts toward higher wavenumbers compared with the pure components, indicating the formation of new hydrogen bonds among hydroxyl, carboxyl, and amino groups from GG, HA, and SPD. Likewise, the C=O and COO^–^ stretching region (1750–1550 cm^–1^) shows partial merging of the KG ester band (∼1735 cm^–1^) with the carboxylate bands of GG and HA, suggesting electrostatic and hydrogen-bond interactions within the polymer network. These spectral modifications support the formation of a physically and ionically cross-linked matrix stabilized by hydrogen bonding and molecular entanglement. Table S1 compiles the characteristic bands and functional group assignments of the pure components to facilitate interpretation. Figure 3 illustrates the structural features of GG/HA (G12) and GG/KG/HA (GK12) hydrogels.

**Figure 3:**
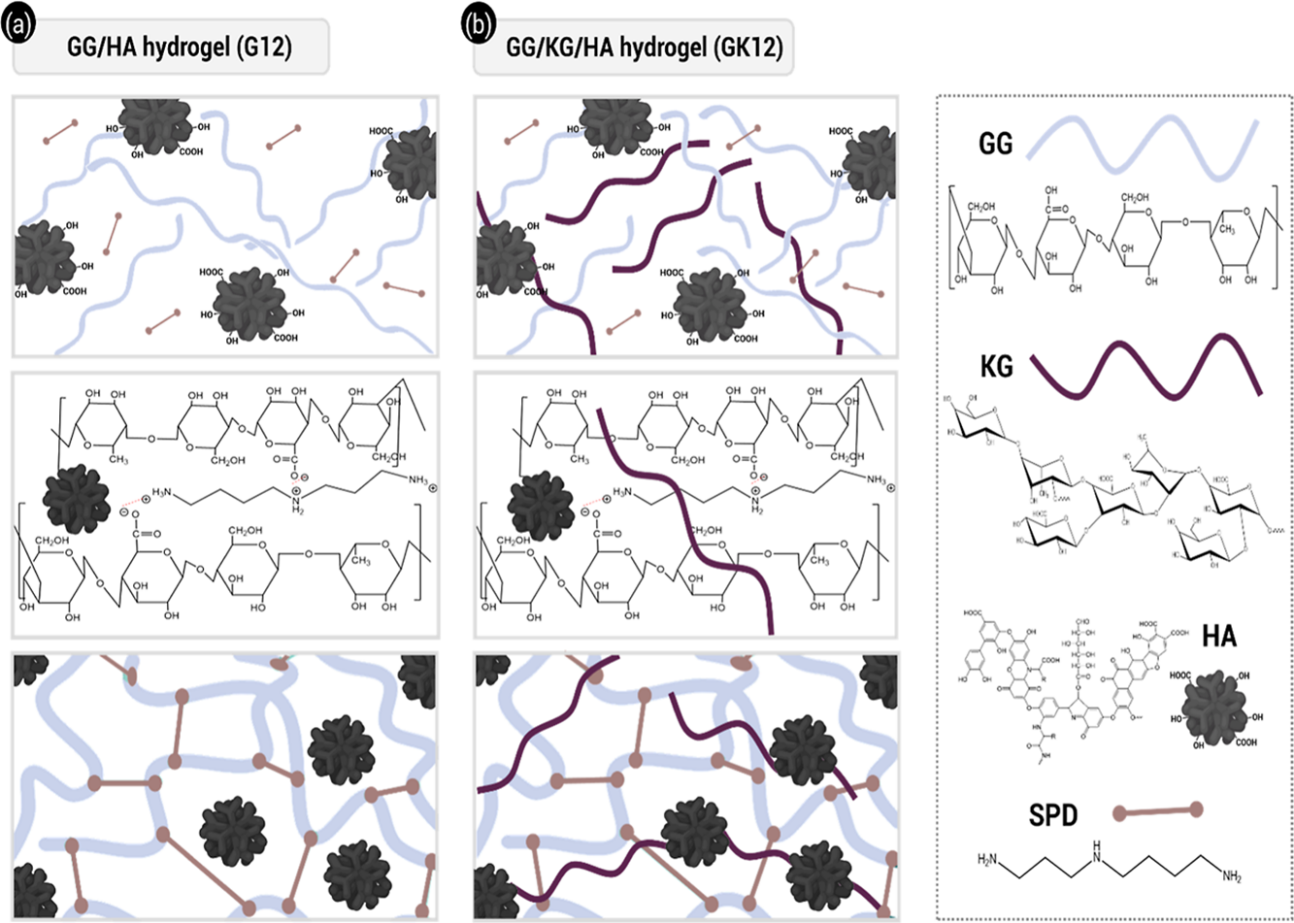
Schematic representation of GG/HA (G12) (a) and GG/KG/HA (GK12) (b) hydrogels.

### TGA Analysis

Figure 4a shows the thermograms of GG, KG, HA, and hydrogel samples of different compositions. Figure 4b shows the temperatures of the maximum rate of weight loss (*T*_max_) of each weight loss step for different materials.

**Figure 4:**
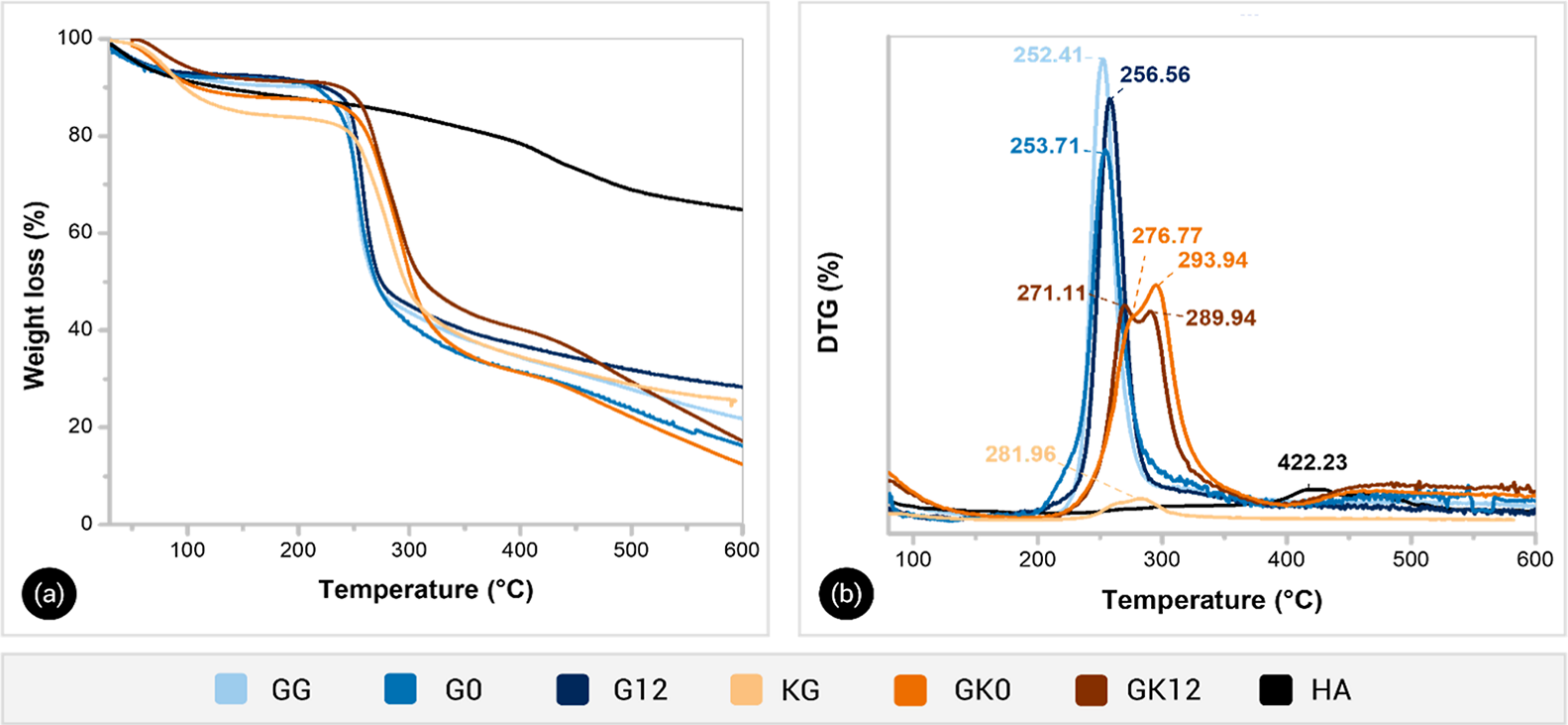
Thermogravimetric (a) and first derivative curves (b) for hydrogel composites and their individual components.

All samples lost mass at temperatures below 100 °C, which was associated with the evaporation of residual moisture. The GG thermogram exhibited a main mass loss (*T*_max_ of 252.41 °C) attributed to main chain degradation and loss of low-weight residues. A final loss above 550 °C was associated with the fragmentation of monomers (monosaccharide units) as previously reported.^13^

The KG thermogram exhibited a weight loss at *T*_max_ of 281.96 °C, consistent with the main chain decomposition profile observed in other polysaccharides, such as gum arabic.^35^

The HA exhibited a multistep degradation process, showing a higher thermal stability than the biopolymers at high temperatures (i.e., more than 50% of HA mass is preserved after heating up to 800 °C), as previously reported.^23,36^

The composite hydrogels showed degradation temperatures comparable to those of their individual counterparts but with distinct two-step profiles. The hydrogels composed of GG/HA exhibited a single main degradation event, with *T*_max_ values close to that of pure GG (253.71 and 256.56 °C for G0 and G12, respectively). In contrast, the GG/KG/HA hydrogels displayed two main degradation steps: the first, occurring at approximately 270 °C (*T*_max_ = 276.77 °C for GK0 and 271.11 °C for GK12), is attributed to the primary decomposition of the GG backbone; and the second, appearing near 290 °C, corresponds to the degradation of KG ester bonds and the partial decarboxylation of HA. These stages occurred at temperatures 20–30 °C higher than those observed for the individual polymers (GG: 252.41 °C, KG: 281.96 °C), evidencing the enhanced thermal resistance of the composite matrix. This improvement in the thermal stability of GG hydrogels was attributed to the mechanical entanglement of KG and HA within the GG framework, as well as the formation of molecular interactions, such as hydrogen bonds, between the H-acceptor and H-donor moieties of the three components.^37,38^

### Mechanical Behavior of Hydrogels

Figure 5a,b show the compressive stress–strain curves of hydrogels and the corresponding Young’s modulus (*E*), respectively. Table 2 summarizes the mechanical parameters at failure. The G0 hydrogel reached a compressive strength of 15.74 ± 0.03 kPa at 26.55 ± 0.12% strain, with *E* around 42 kPa. This response is consistent with the rigid behavior of ionotropically cross-linked GG gels.^39^ Adding HA stiffened and strengthened the network: G12 sustained 18.74 ± 1.73 kPa, failed at 28.2 ± 1.27%, and exhibited *E* around 60 kPa, indicating reinforcement by additional interactions between HA and GG chains. This reinforcing effect of humic substances is in line with observations in other biopolymer-based hydrogels, where adding moderate amounts of HA improved the mechanical stability of material.^38^

**Figure 5:**
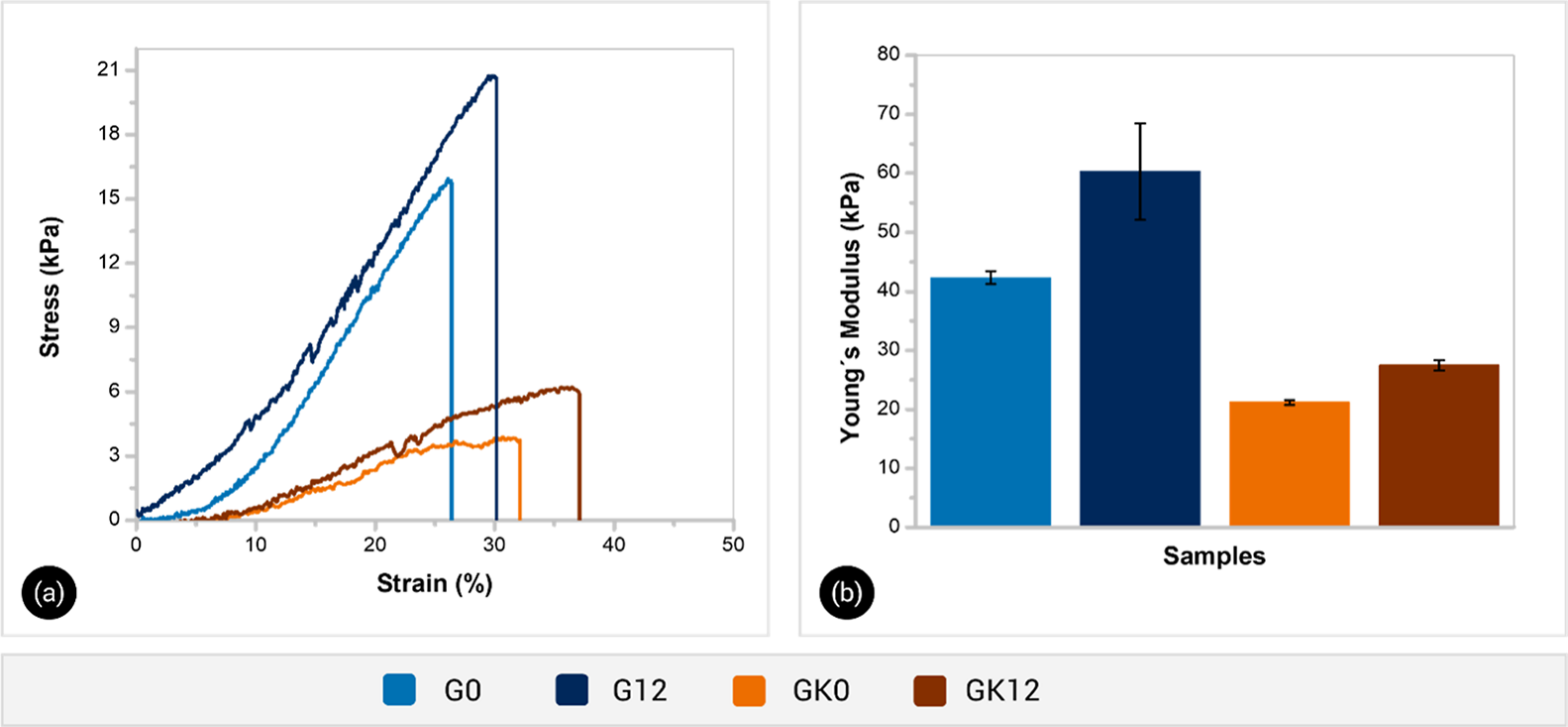
Representative compressive stress–strain curves (a) and Young’s modulus (b) for hydrogels.

**Table 2:** Compression Properties at Failure of Composite Hydrogels

sample	compressive strength (kPa)	failure strain (%)
G0	15.74 ± 0.03	26.55 ± 0.12
G12	18.74 ± 1.73	28.2 ± 1.27
GK0	3.86 ± 0.16	32.68 ± 0.49
GK12	5.71 ± 0.53	37.71 ± 0.38

In contrast, GG/KG/HA hydrogels were softer but more deformable over the entire strain range. GK0 showed a compressive strength of 3.86 ± 0.16 kPa at 32.7% strain and *E* = 21 kPa, 75% less than G0 strength and roughly half its modulus. GK12 followed the same trend, with 5.71 ± 0.53 kPa and *E* = 27.5 kPa, both well below than G12. These results indicate that incorporating KG into the hydrogel reduces its mechanical strength while increasing its ductility. This effect can be attributed to two main factors: (i) the presence of KG chains during gelation likely interferes with the ionotropic cross-linking of GG with spermidine, resulting in a lower cross-link density; and (ii) the GG/KG/HA formulations contain a lower absolute amount of GG, GK0 and GK12 had roughly half the GG content of G0 and G12, with KG occupying the remainder. Since GG forms the rigid cross-linked backbone, reducing its proportion (and partially substituting it with a less cross-linkable polysaccharide) inherently diminishes the hydrogel’s overall strength. Thus, both the cross-link density and the effective network polymer concentration are reduced in GG/KG/HA hydrogels, explaining their substantially lower compressive moduli and strengths.

An effective soil conditioner must combine sufficient compressive strength to maintain integrity during mixing and surface burial, with high ductility to withstand soil compaction and wet–dry swelling cycles (1–50 kPa).^40^ Among our formulations, G12 was the strongest yet comparatively brittle, whereas GK0 was highly deformable but mechanically weak. In contrast, GK12 achieved the most favorable balance: HA provides reinforcement while KG imparts extensibility, yielding a network that best meets the mechanical requirements for agricultural applications.

### SEM Analysis

Figure 6 shows SEM micrographs of cross sections of freeze-dried G0, G12, GK0, and GK12 hydrogels at different magnifications. A well-defined network-shaped porous structure is observed in all samples, consistent with the morphology of SPD cross-linked GG hydrogels.^29^ For all hydrogels, a heterogeneous pore size distribution is observed. In both systems (with or without GK), the incorporation of HA increases the pore dimensions (Figure 6b,f,d and h), compared to those samples that do not contain it (Figure 6a,e,c and g).

**Figure 6:**
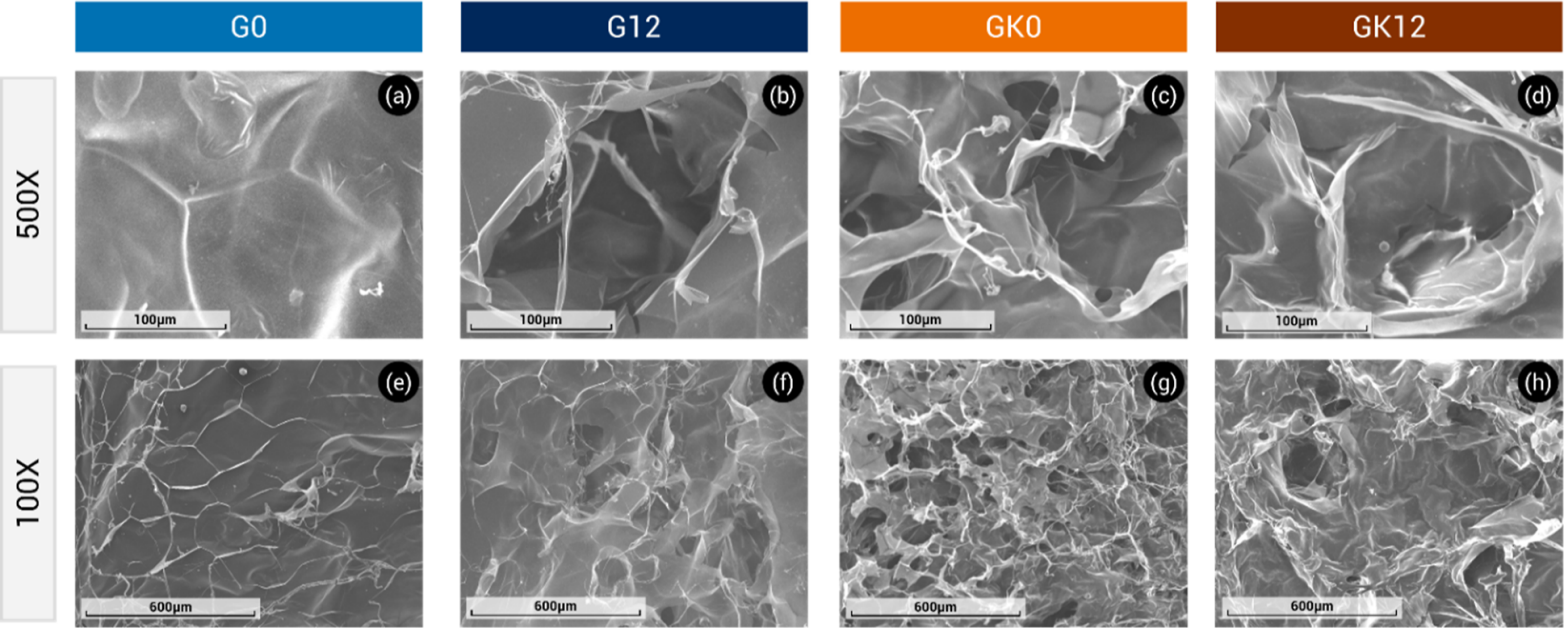
SEM micrographs of cross sections of G0 (a,e), G12 (b,f), GK0 (c,g), and GK12 (d,h) hydrogels at 500X (a–d) and 100X (e–h) magnifications.

Quantitative pore size analysis (Figure S1) confirmed these visual observations. The average pore diameters were 78 ± 15 μm (G0), 92 ± 18 μm (G12), 64 ± 14 μm (GK0), and 70 ± 16 μm (GK12). These results suggest that the incorporation of HA leads to larger pores, likely because humic macromolecules act as spacers within the polymeric matrix. Conversely, the addition of KG produces slightly smaller pores, suggesting that KG chains occupy free volume within the GG network, yielding a more compact structure.

These morphological differences align with the mechanical characteristics discussed in the previous section. Hydrogels containing HA (G12 and GK12) displayed reinforced architecture with thicker pore walls, while the incorporation of KG resulted in softer, more compliant matrices featuring thinner and more flexible walls. This microstructural evidence supports the complementary roles of HA and KG in modulating both the architecture and mechanical behavior of GG-based hydrogels, ensuring a balance between structural integrity and flexibility suitable for agricultural applications. The larger pore diameters observed in HA-containing samples (G12 and GK12) correspond to improved deformability and moderate stiffness, whereas the compact pore networks of G0 and GK0 are consistent with increased rigidity and reduced flexibility. These results highlight the influence of microstructural features on the bulk mechanical response.

### Swelling Measurements

The swelling profiles were obtained for each hydrogel at room temperature in deionized water and Yaqui valley soil extract. Figure 7 shows the swelling kinetics of GG/HA and GG/KG/HA hydrogels in both media and the equilibrium swelling degrees are summarized in Table 3. A rapid swelling occurred for all samples within the first hour due to the surface hydrophilicity and capillarity of pores of materials.

**Figure 7:**
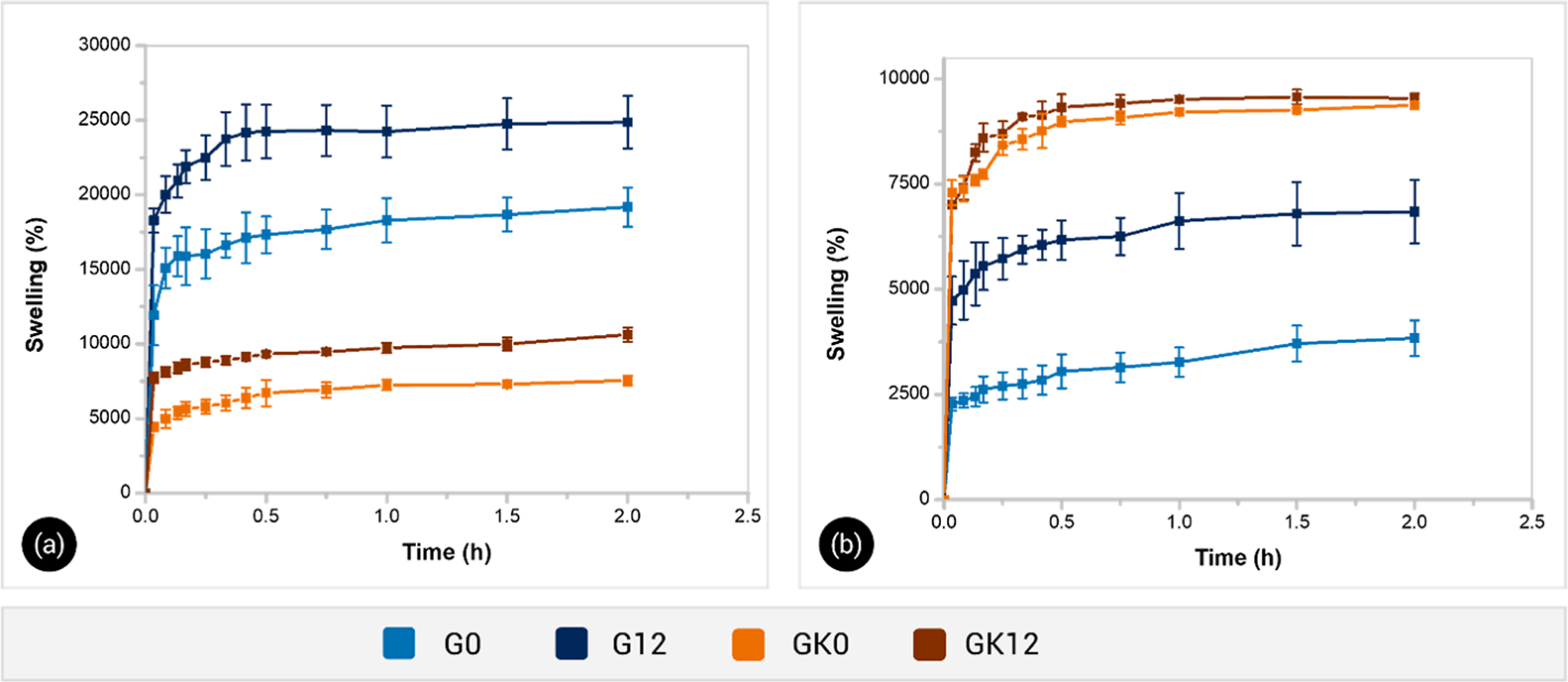
Swelling kinetics of composite hydrogels in deionized water (a) and soil extract (b) at 25 ± 1 °C.

**Table 3:** Equilibrium Swelling Degree of GG/HA and GG/KG/HA Hydrogels in Deionized Water and Soil Extract at 25 ± 1 °C

sample	deionized water (%)	soil extract (%)
G0	19,170 ± 1,314	3840 ± 420
G12	24,866 ± 1768	6844 ± 750
GK0	7560 ± 315	9380 ± 33
GK12	10,633 ± 462	9540 ± 117

In deionized water, the maximum degree of swelling reached by formulation G0 was 19,170 ± 1314%. However, when HA was added (G12), the equilibrium swelling degree increased to 24,866 ± 1768% (Figure 7a). HA of soils is a multicomponent system with an amphiphilic nature. Its functional groups include hydrophilic moieties such as carboxylic, phenolic, enolic–OH, amino, and sulfhydryl groups. These highly polar groups form strong hydrogen bonds with water molecules. Additionally, its ionized groups in an aqueous medium (pH 7 ± 0.2) increase electrostatic repulsion forces, thereby enhancing the swelling capacity of the hydrogel.^41^

Samples containing KG and a lower GG content exhibited a reduced swelling capacity in deionized water compared to G0 and G12, reaching 7560 ± 315% and 10,633 ± 462% for GK0 and GK12, respectively, after 2 h. GG is an anionic linear polysaccharide with carboxylic groups that ionize in water, forming hydrogen bonding and electrostatic repulsion interactions. In contrast, KG has a complex, partially acetylated structure (∼8% acetyl groups by weight). These acetyl groups reduce the affinity of KG for water molecules compared to GG, thereby diminishing the swelling capacity of hydrogels. This swelling behavior was also consistent with the SEM observations.

On the other hand, the swelling level of all samples was slightly lower in soil extract (Figure 7b) compared to deionized water. At high ionic strength, the increased ion concentration raises the osmotic pressure of the hydrogel, causing water to desorb from the network.^30,42^ Moreover, the ionized groups of polysaccharides were able to interact with the counterions in the absorption medium, reducing the availability of hydration sites on polymer chains and consequently lowering the diffusion rate of water molecules.

Interestingly, the swelling capacity of hydrogels in the Yaqui Valley soil extract significantly improved with the addition of KG, contrary to their behavior in deionized water. Both GK0 and GK12 samples reached swelling levels close to 10,000% (9380 ± 33% and 9540 ± 117%, respectively), while samples G12 and G0 exhibited swelling levels of 6844 ± 750% and 3840 ± 420%, respectively. It is suggested that the charged and highly polar pendant groups of KG chains hindered the interaction between GG and extract ions, thereby enhancing the absorption of water molecules into the network, which is a key characteristic for hydrogels intended in agriculture applications.

A critical aspect for hydrogels intended for agricultural use is the rate at which they absorb water and reach swelling equilibrium. All formulations exhibited a rapid initial uptake, absorbing most of the water within the first hour of immersion, and effectively reached equilibrium within ∼2 h for both GG/HA and GG/KG/HA hydrogels.

Achieving near-equilibrium swelling within 1–2 h is both acceptable and advantageous for agricultural superabsorbents. Following irrigation or rainfall, soil water availability is transient, as percolation and runoff may occur within minutes to hours. Thus, a hydrogel that swells rapidly can sequester moisture before it drains beyond the root zone or evaporates and then release it gradually over subsequent days to buffer plants against short-term drought. Consistent with this requirement, reported swelling half-times for superabsorbent polymers typically range from minutes to a few hours, depending on formulation, cross-link density, and particle size.^43,44^

The swelling capacity of both GG/HA and GG/KG/HA hydrogels is comparable to values reported for soil-conditioning hydrogels. Synthetic polyacrylate-based hydrogels typically absorb ∼300–500 g g^–1^ in deionized water, and up to ∼500–800 g g^–1^ for premium diaper-grade poly­(acrylic acid) formulations.^45,46^ However, their absorbency decreases sharply in saline media; for instance, in 0.9% NaCl (∼15,000 μS cm^–1^), (which is considerably higher than the ionic strength of the Yaqui valley soil extract), polyacrylates often swell to <100 g g^–1^ and may drop to 20–50 g g^–1^ under higher salinity.^45^

Natural polymer hydrogels generally exhibit equilibrium swelling of a few hundred to several thousand percent; for example, chitosan-graft systems reach ∼2500% in water, starch-based systems ∼1500–4500%, and guar gum/acrylate copolymers ∼3650–5300% in distilled water.^4^ In our study, GG/HA and GG/KG/HA hydrogels exceeded these benchmarks in pure water (G12 ∼ 25,000%), whereas a previously reported GG/konjac-glucomannan hydrogels, prepared via thermal gelation with Ca^2+^ ions, swelled only ∼300% under neutral pH.^16^ More importantly, in soil extract the GG/KG/HA hydrogels maintained high swelling (GK0 and GK12 ∼ 10,000%), indicating enhanced salt tolerance and thereby promoting water retention within the root zone.

Considering both swelling capacity and kinetics, GG/HA and GG/KG/HA hydrogels perform within the same order of magnitude as conventional polyacrylate superabsorbent under ionic/soil-like conditions, while offering the additional advantages of biodegradability and potentially improved soil integration, supporting their use for drought mitigation in agriculture.

In addition, the long-term stability of the hydrogel network was assessed. A lyophilized GK12 sample stored at room temperature for ∼20 months retained 92.5% of its original swelling capacity when rehydrated in soil extract (8676 ± 182% vs 9540 ± 117% for the fresh sample). This confirms the structural integrity of the GG/KG/HA matrix during extended ambient storage, supporting its practical applicability (Figure S2).

### Water Retention Study in Soil

Water evaporation from soil is influenced by ambient conditions such as air temperature, relative humidity, and the capacity of the soil to retain water.^31^ The water retention capacity of soils produces positive effects on the seedling survival rates and plant growth.^47,48^ Hydrogels modify soil structure by reducing drainage pores and retaining water, which can decrease the evaporation of soil water bound to the hydrogel, thereby reducing water loss to the atmosphere.^10^

Figure 8 shows the WER values of soils containing GG-based and commercial (WSH) hydrogels, as well as hydrogel free-soil, over 15 days. The presence of 0.5 wt % GG hydrogels enhanced water retention, demonstrating better soil moisture preservation compared to the WSH sample. After 15 days, WER values decreased in the following order: WSH (96.47%), pure soil (control) (93.97%), G0 (90.35%), G12 (88.47%), GK0 (87.89%), and GK12 (85.79%). This tendency indicates that the GG/KG/HA hydrogels, particularly GK12, retain water more effectively in soil than the other formulations, consistent with their enhanced performance observed in swelling tests in soil extract. Additionally, the commercial hydrogel began to crack upon moisture loss, exhibiting a greater tendency for dehydration compared to pure soil control.

**Figure 8:**
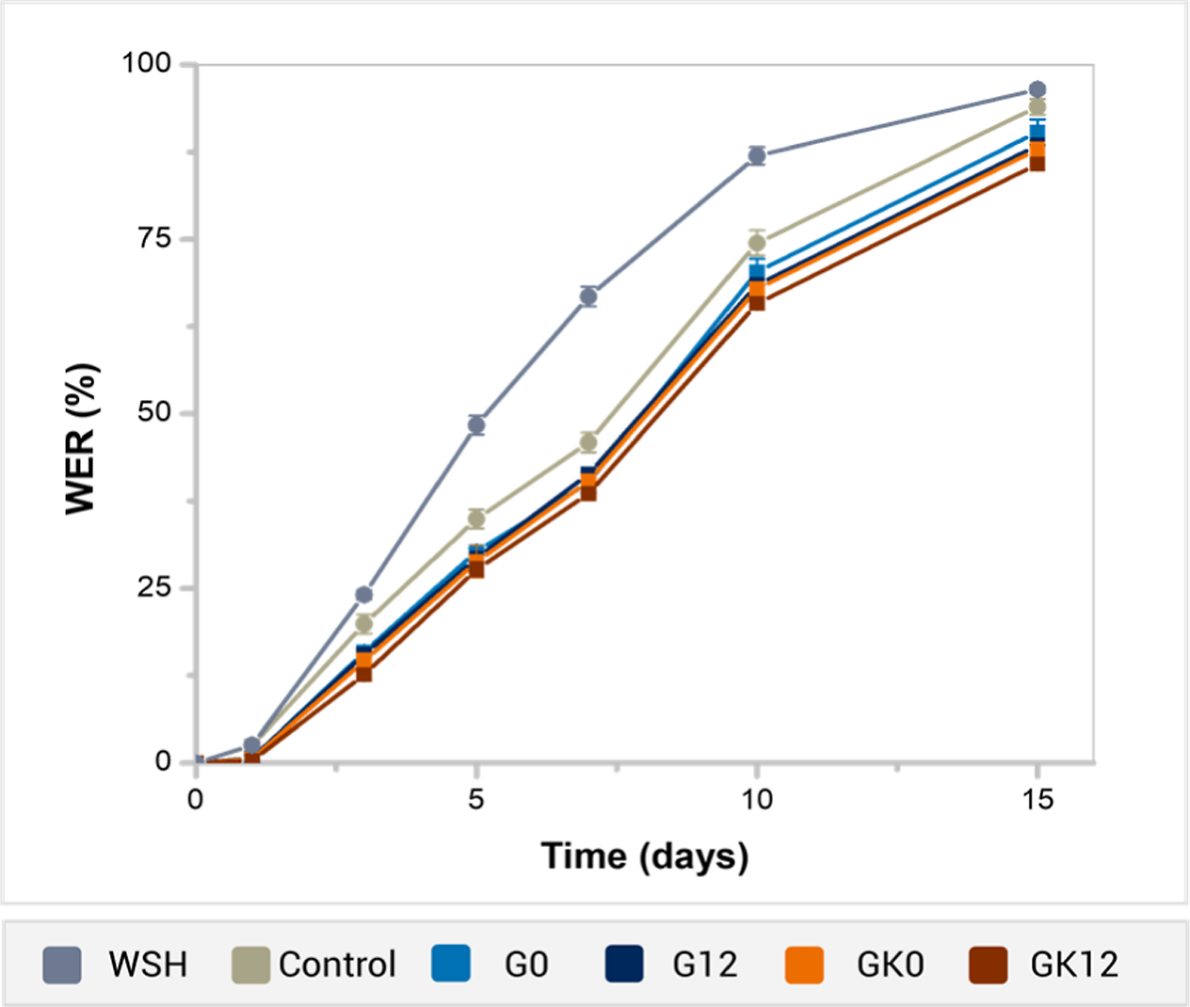
Water evaporation ratio of pure soil and soil containing GG-based hydrogels and a commercial sample (WSH).

### Biodegradation Test

The biodegradation of hydrogels in soil is a desirable property to ensure their safe use in agricultural applications. The biodegradability of GG-based hydrogels was studied by monitoring the weight loss of the samples in an aqueous extract of Yaqui valley soil at room temperature (25 ± 1 °C) over 30 days.

KG-containing samples exhibited greater weight loss than hydrogels without KG throughout most of the study period (Figure 9). After 30 days, the highest weight losses (39.15–40.92%) were observed for GK0, GK12, and G0 samples, with only marginal differences among them. In contrast, the G12 hydrogels showed the lowest weight loss (33.52%). These results suggest that the KG/GG ratio positively influences the degradation rate of hydrogels under biologically active soil conditions.

**Figure 9:**
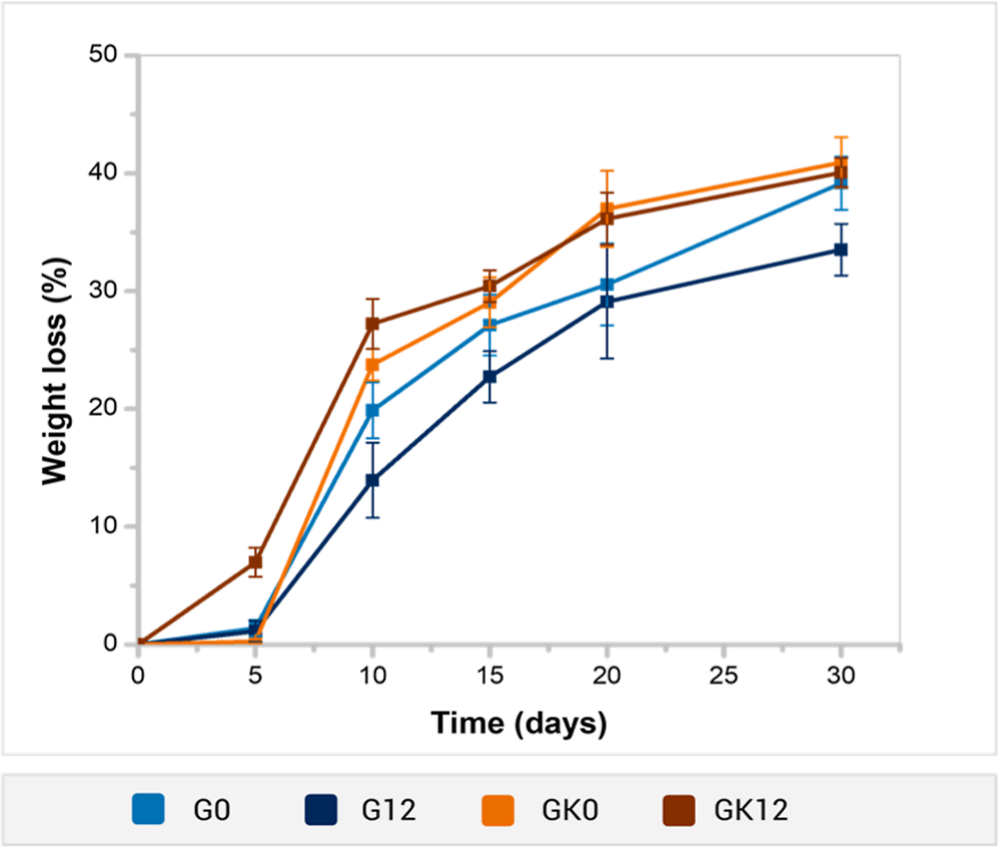
Biodegradation study of GG-based hydrogels in aqueous extract of Yaqui Valley soil for 30 days.

In comparison with previously reported systems, similar GG-based hydrogels cross-linked with poly­(acrylic acid) exhibited rapid biodegradation, reaching approximately 87% mass loss within 3 weeks under soil and composting conditions.^2^ In contrast, GG macro-beads prepared without chemical modification remained essentially intact after 40 days under comparable simulated soil burial conditions, whereas alginate and agar analogues underwent substantial microbial decomposition.^49^ Other natural polysaccharide hydrogels, such as chitosan/carboxymethyl cellulose/silk fibroin composites, have shown markedly faster degradation (∼78% mass loss within 2 weeks soil burial).^50^

These remarks indicate that the biodegradation rate of polysaccharide-based hydrogels is strongly influenced by their chemical composition, degree of cross-linking, and network architecture. The GG/KG/HA hydrogels developed in this study exhibited an intermediate degradation profile, maintaining structural integrity for at least one month under soil-like conditions while progressively undergoing microbial breakdown. This behavior provides a favorable balance between functional persistence and environmental compatibility, making them suitable for agricultural applications.

Given that the hydrogels are composed of naturally derived materials, their degradation may contribute to soil microbial communities by serving as nutrient sources.^51^ The breakdown products of polysaccharides often possess biostimulant activity and, in some cases, are more effective than their native forms. For instance, partially hydrolyzed GG has been reported to enhance vegetative growth, flowering, and salt stress tolerance in pansy plant.^22^

During the biodegradation process, the GG/KG/HA hydrogels are expected to release natural byproducts such as oligosaccharides, uronic acids, amino-rich fragments from SPD cross-links, and low-molecular-weight humic derivatives. These compounds are inherently nontoxic and may act as carbon sources for soil microorganisms or biostimulants for plant roots, supporting nutrient cycling and microbial diversity while minimizing ecological risks in agricultural environments.^51^

### Effects of Hydrogel on Plant Growth

The absence of phytotoxicity is a fundamental requirement for using any polymeric material as a component of the growing medium of plants. In the assays, 100% of wheat seeds germinated in all hydrogel samples, whereas only 65% of the seeds germinated in the PDA control. According to some reports, a germination rate above 60% is accepted as an indicator of nonphytotoxicity.^6^ From this point of view, all hydrogel samples used in the study are safe for plant germination. GG has been evaluated as a support material for agricultural applications, especially for the propagation of plant tissues.^52^

Figure 10 shows the chlorophyll concentrations in seedlings after 10 days of growth. In all hydrogel samples, the chlorophyll concentration in wheat seedlings increased significantly compared to those grown on PDA. Specifically, chlorophyll concentrations increased from 110.5 ± 11.83 μM·m^–2^ in the control to 201.86 ± 11.51, 223.94 ± 5.56, 256.0 ± 7.70, and 326.88 ± 9.85 μM·m^–2^ for G0, G12, GK0, and GK12, respectively. The addition of HA and KG to the hydrogels enhanced the chlorophyll levels, with the highest concentration observed in the GK12 sample. Previous works have shown that HA significantly promotes chlorophyll content in leaves, as observed in corn crops,^53^ along with its beneficial effect on moisture retention and salinity mitigation in crop soils.^54^ On the other hand, KG-based materials have been proposed as gelling agents instead of conventional agar for micropropagation and regeneration of lemon plants (*C. jambhiri* Lush.).^21^ To the best of our knowledge, no prior studies have reported a positive effect of KG on chlorophyll content or overall biomass in agriculturally relevant crops. Although KG has been previously explored as a gelling agent for in vitro plant tissue culture,^21^ its application as a bioactive component in soil amendments or hydrogels for crop production remains largely unexplored. This study therefore provides novel evidence of KG potential to enhance plant physiological performance, particularly in terms of chlorophyll accumulation and biomass production.

**Figure 10:**
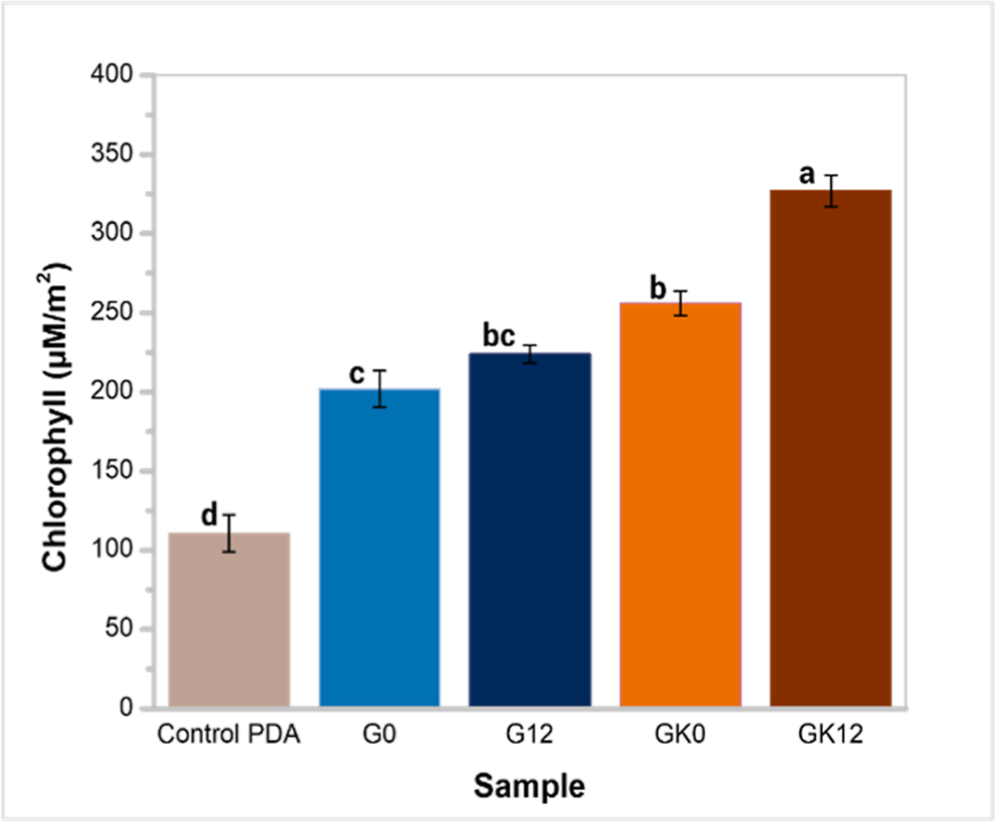
Chlorophyll content in wheat (*T. aestivum* L. var. Borlaug 100) grown on PD agar (control) and hydrogel samples. Different letters in the columns indicate significant differences at *P* < 0.05.

Figure 11 shows the average fresh and dry weights of roots and aerial parts of wheat plants that grew in the presence of hydrogel samples and the control (PDA). Statistical analysis revealed significant differences in the biomass yield of both roots and aerial parts depending on the sample used. Fresh root weights increased from 33.3 ± 6.1 mg·plant^–1^ (control) to 73.0 ± 8.3 mg·plant^–1^ (GK12), while dry root weights increased from 6.1 ± 1.3 mg·plant^–1^ (control) to 11.3 ± 0.7 mg·plant^–1^ (GK12). Similar trends were observed for the aerial parts, where fresh and dry weights rise from 72.3 ± 15.0 and 12.2 ± 1.1 mg·plant^–1^ in the control to 214.4 ± 26.1 and 23.7 ± 2.0 mg·plant^–1^ in GK12, respectively. These quantitative results, along with the increased chlorophyll accumulation, confirm that the addition of HA and KG to GG-based hydrogels contributes to improved wheat growth performance, likely due to enhanced water retention, greater nutrient availability, and possible biostimulant effects associated with the hydrogel composition.

**Figure 11:**
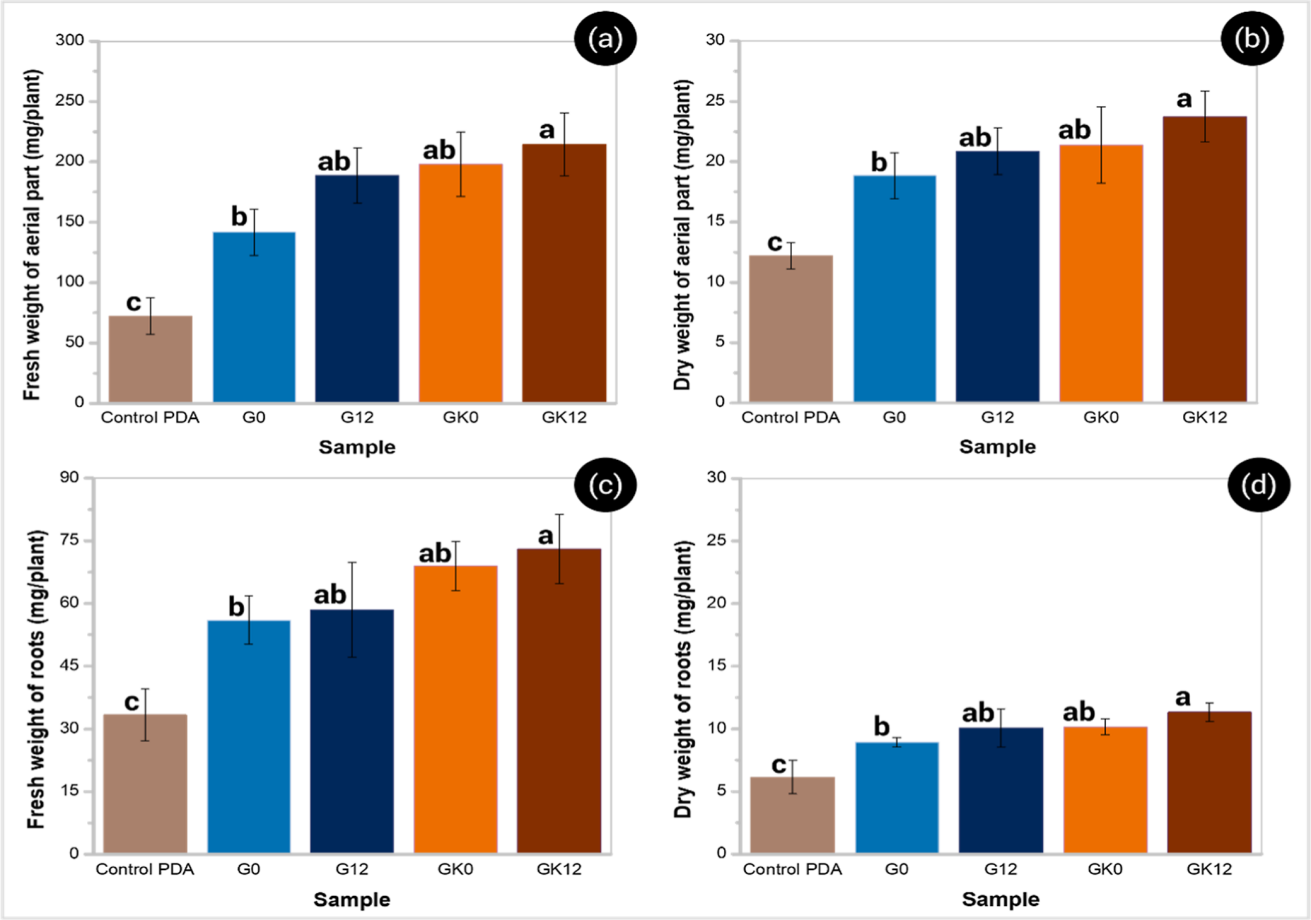
Effect of composite hydrogels on fresh and dry weight of aerial parts (a,c) and roots (b,d) of wheat (*T. aestivum* L. var. Borlaug 100). Different letters in the columns indicate significant differences at *P* < 0.05.

## Conclusions

GG-based hydrogels cross-linked with SPD, with and without the incorporation of KG and HA, were successfully developed and characterized for potential agricultural applications. All hydrogel formulations showed a heterogeneous, interconnected porous morphology, with pore dimensions influenced by the presence of HA and KG. Mechanical testing revealed that the inclusion of KG improved the ductility of the hydrogels, while HA enhanced their compressive strength. All samples showed rapid swelling and high-water absorption capacity in deionized water, particularly those containing HA. Notably, hydrogels with KG exhibited greater swelling levels in soil extracts from Yaqui valley, suggesting improved performance under real environmental conditions. The hydrogels degraded gradually over 30 days in biologically active soil, with the highest and lowest weight loss observed in GK0 and G12, respectively. None of the hydrogel formulations exhibited phytotoxicity in a *T. aestivum* L. var. Borlaug 100 growth test, achieving 100% germination in contrast to the 65% observed in the control. Significant increases in chlorophyll content and biomass production were recorded in plants grown with hydrogels, particularly in formulations containing both KG and HA. Importantly, a lyophilized GK12 hydrogel stored at room temperature for approximately 20 months retained 92.5% of its original swelling capacity when rehydrated in soil extract, confirming the long-term structural integrity and practical storage stability of the GG/KG/HA matrix. This work demonstrated that the GG/KG-based hydrogels combined with HA possess suitable physicochemical, mechanical, and biological properties for sustainable agricultural applications aimed at improving soil health and promoting plant growth. Although the experiments were conducted using a clay–loam soil extract from the Yaqui valley, the mechanisms responsible for swelling, water retention, and biodegradation are expected to operate similarly in other soil types. Future long-term field trials or assessments of GG/KG/HA hydrogels under repeated wet–dry cycles are important for evaluating functionality and resilience of materials under real agricultural conditions.

## Supplementary Material



## Notes

FTIR band assignments of the formulation components, pore size distribution of the hydrogels, and swelling behavior of the GK12 hydrogel in soil extract (PDF)
