# Supplementary material for: Novel Polysaccharide Hydrogels Enriched with Humic Acid for Sustainable Agricultural Applications
Source: ACS Omega. 2025 Dec 16;10(51):63189–201. doi: 10.1021/acsomega.5c09496 (PMC12756770; doi:10.1021/acsomega.5c09496)
Supplement: Supplementary file 1 [file ao5c09496_si_001.pdf]

Supporting Information

Novel polysaccharide hydrogels enriched with humic acid for sustainable agricultural applications

AUTHOR NAMES

Ana V. Torres-Figueroa[1,4], Sergio de los Santos-Villalobos[2], Dora E. Rodríguez-Félix[1], Gerardo Valenzuela-Hernandez[5], Sergio F. Moreno-Salazar[3], Cinthia J. Pérez-Martínez[4], Andrés Ochoa-Meza[3*], Teresa del Castillo-Castro[1*]*

AUTHOR ADDRESS

[1] Departamento de Investigación en Polímeros y Materiales, Universidad de Sonora, Hermosillo 83000, Mexico.

[2] Laboratorio de Biotecnología del Recurso Microbiano, Departamento de Ciencias Agronómicas y Veterinarias, Instituto Tecnológico de Sonora, 5 de Febrero 818 Sur, Colonia Centro, Obregón 85000, Mexico.

[3] Departamento de Agricultura y Ganadería, Universidad de Sonora, Carr. Bahía de Kino, Km. 21. Apartado Postal 305, Hermosillo 83323, Sonora, Mexico.

[4] Departamento de Ciencias Químico Biológicas, Universidad de Sonora, Hermosillo 83000, Mexico.

[5] Departamento de Investigación en Física, Universidad de Sonora, Hermosillo 83000, Mexico.

*Corresponding authors email: anavaleria.torres@unison.mx, andres.ochoa@unison.mx, teresa.delcastillo@unison.mx

Table S1. Assignment of FTIR bands.

**Sample**	**Wavenumber (cm^{-1})**	**Functional groups**
**GG**	3506-3117	(O-H)
	2870	(C-H)
	1604, 1402	(COO^-)
	1015	(C-O-C)
**KG**	3567-3110	(O-H)
	2966	(C-H)
	1735, 1226	(C=O)
	1605, 1371	(COO^-)
**HA**	3385	(O-H)

2927	(C-H)
1690	(COO^-)
1585	(C=C)
1394	(COO^-)
1106	(CO)
1035	(C-N)

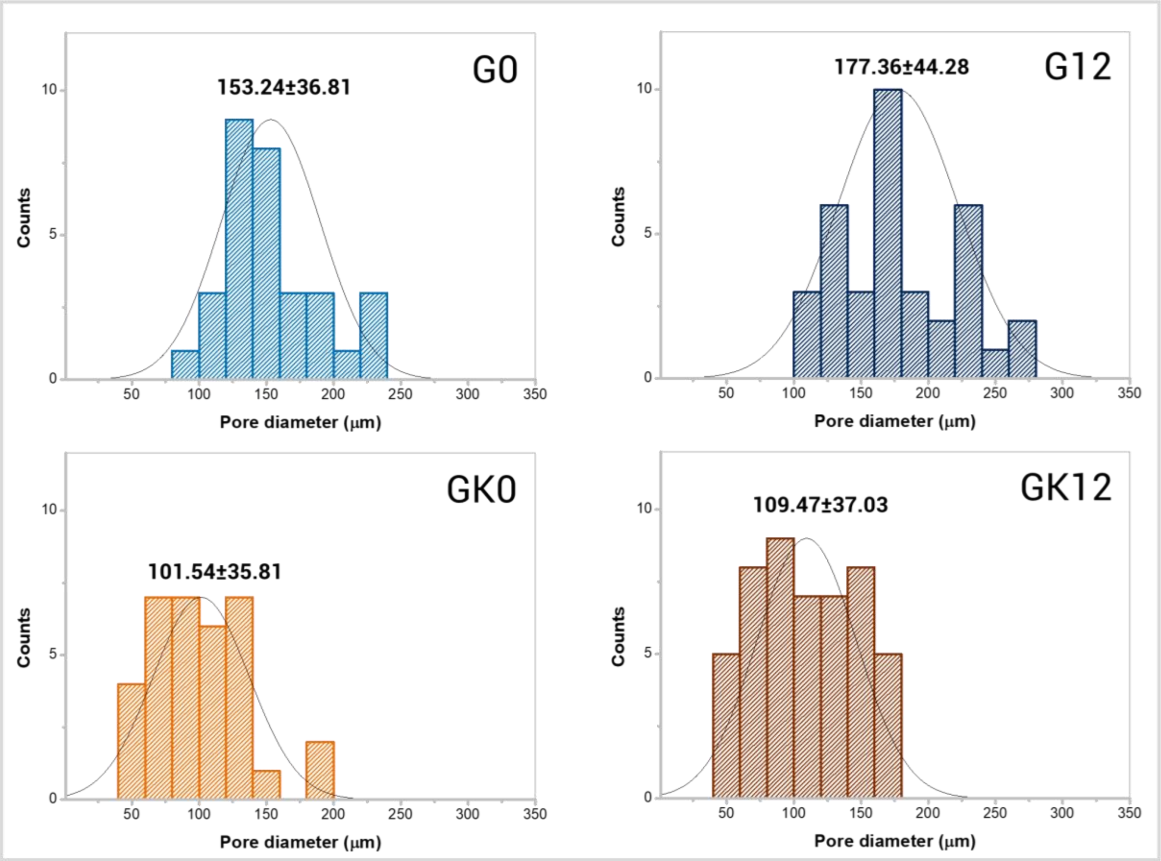

Figure S1. Pore size distribution.

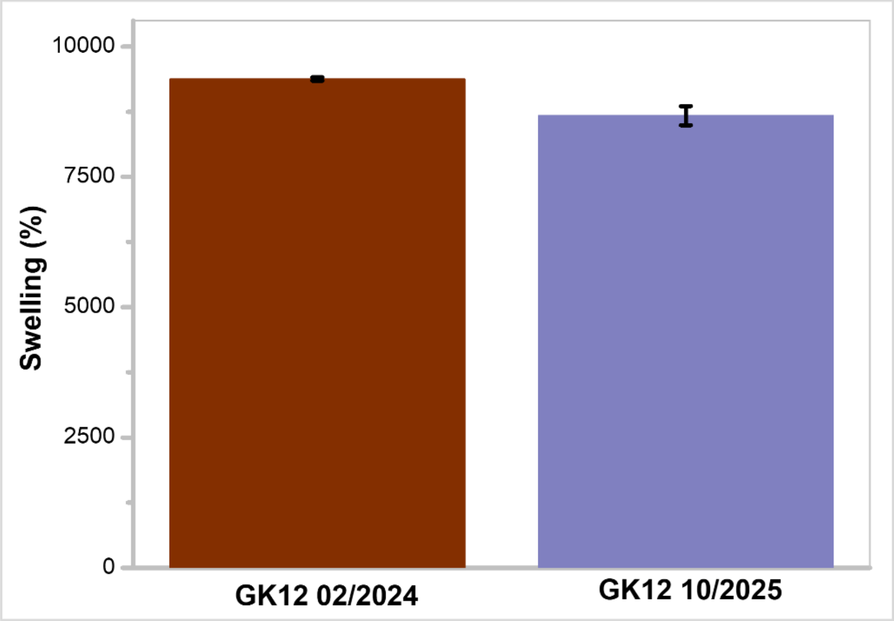

Figure S2. Swelling of GK12 in soil extract for a freshly prepared sample (02/2024) and after ~20 months of room-temperature storage (10/2025). Values are mean ± SD (n = 3).
